# Deciphering complex genome rearrangements in *C. elegans* using short-read whole genome sequencing

**DOI:** 10.1038/s41598-021-97764-9

**Published:** 2021-09-14

**Authors:** Tatiana Maroilley, Xiao Li, Matthew Oldach, Francesca Jean, Susan J. Stasiuk, Maja Tarailo-Graovac

**Affiliations:** 1 Departments of Biochemistry, Molecular Biology and Medical Genetics, Cumming School of Medicine, University of Calgary, Calgary, AB T2N 4N1 Canada; 2 Alberta Children’s Hospital Research Institute, University of Calgary, Calgary, AB T2N 4N1 Canada

**Keywords:** Computational biology and bioinformatics, Genetics

## Abstract

Genomic rearrangements cause congenital disorders, cancer, and complex diseases in human. Yet, they are still understudied in rare diseases because their detection is challenging, despite the advent of whole genome sequencing (WGS) technologies. Short-read (srWGS) and long-read WGS approaches are regularly compared, and the latter is commonly recommended in studies focusing on genomic rearrangements. However, srWGS is currently the most economical, accurate, and widely supported technology. In *Caenorhabditis elegans* (*C. elegans*), such variants, induced by various mutagenesis processes, have been used for decades to balance large genomic regions by preventing chromosomal crossover events and allowing the maintenance of lethal mutations. Interestingly, those chromosomal rearrangements have rarely been characterized on a molecular level. To evaluate the ability of srWGS to detect various types of complex genomic rearrangements, we sequenced three balancer strains using short-read Illumina technology. As we experimentally validated the breakpoints uncovered by srWGS, we showed that, by combining several types of analyses, srWGS enables the detection of a reciprocal translocation (*eT1*), a free duplication (*sDp3*), a large deletion (*sC4*), and chromoanagenesis events. Thus, applying srWGS to decipher real complex genomic rearrangements in model organisms may help designing efficient bioinformatics pipelines with systematic detection of complex rearrangements in human genomes.

## Introduction

Structural variations (SVs) are genomic rearrangements such as copy number alterations, inversions, and translocations. More complex events, known as chromoanagenesis, combine a cascade of chromosomal rearrangements^1^. Over the past few years, structural variants and complex genomic rearrangements have been implicated in various phenotypes: cancer^2,3^, rare disorders^4–9^ and common diseases^10^ in humans, reproduction traits in pigs^11^, virulence traits in plant pathogenic fungi^12^, local adaptation in maize^13^, and behavior in *Caenorhabditis elegans* (*C. elegans*)^14^. However, the technologies and methods used to identify SVs and complex rearrangements are still multifaceted and no approach has yet been recognized as standard. Short-read and long-read whole genome sequencing (WGS) technologies, as well as their respective tools and pipelines, are often assessed and compared in their ability to detect structural variants and complex rearrangements^15–21^. The read length of short-read technologies is often reported as a limitation for detecting larger and more complex events^22^. Meanwhile, long-read sequencing and linked-reads approaches are gaining popularity^23–25^, especially when the analysis of short-read sequencing data fails to uncover SVs and complex rearrangements of interest^26,27^. Here, we focused on short-read WGS of *C. elegans* strains known to harbor SVs and show that short-read WGS provides enough data to decipher SVs of various types and complex genomic rearrangements in these genomes when tailored workflows are used.

In *C. elegans*, SVs and complex rearrangements have been used for decades to balance large parts of the genome by suppressing crossover events and maintaining heterozygosity. It facilitates the investigation of lethal mutations, the construction of new strains, and the screening of mutations^28^. While some balancers are spontaneous, like the reciprocal translocation *nT1(IV;V)*^28^, most were created via random mutagenesis processes, such as X-ray mutagenesis, chemical mutagens (acetaldehyde, ENU, EMS), gamma irradiation, and more recently by using CRISPR-Cas9 methods^29,30^. For most of the mutagen-induced balancers, the implicated chromosomal rearrangements are uncharacterized at the molecular level (i.e., precise genomic position and nature of the rearrangement are unknown. Thus, *C. elegans* balancers constitute an interesting source of various genomes and complex genomic rearrangements to assess the ability of short-read PCR-free WGS Illumina technologies and tailored bioinformatics workflows to detect and characterize complex structural variants. Here, we sequenced the genomes of three *C. elegans* balancers, ranging from a well-characterized SV [*eT1(III;V)*, a reciprocal translocation] to an uncharacterized and molecularly unknown balancer [*sC4* (BC4586)]. Beyond the successful proof-of-concept detection of *eT1(III;V)*, we deciphered the structure and genomic positions of *sDp3* and *sC4*, as well as additional rearrangements not previously known to exist in the balancer strains selected for this study (BC4586, BC986, and VC109).

In our study, we found that short-read WGS datasets can be used to detect, identify, and characterize SVs and complex genomic rearrangements in *C. elegans* genomes. The knowledge gained from the analytical methods used on *C. elegans* balancers may help optimize detection and characterization of complex variants in humans using short-read WGS.

## Results

### Short-read WGS can be used to detect homozygous and heterozygous reciprocal translocations

The strains BC986 and VC109 carry the reciprocal translocation *eT1(III;V).* In *C. elegans*, the reciprocal translocation *eT1(III;V)* balancer has been well studied and it is described as balancing LGV, from the left chromosome end through *unc-23*, and LGIII, from the right end to *unc-36*^31^. Its genomic breakpoints were more recently localized in the second intron of *unc-36* on LGIII and between *rol-3* and *unc-42* on LGV^32^. Therefore, we first focused our efforts on retrieving *eT1(III;V)* breakpoints, to assess the ability of short-read WGS to decipher reciprocal translocations as a proof of concept for our approach.

**Figure 1:**
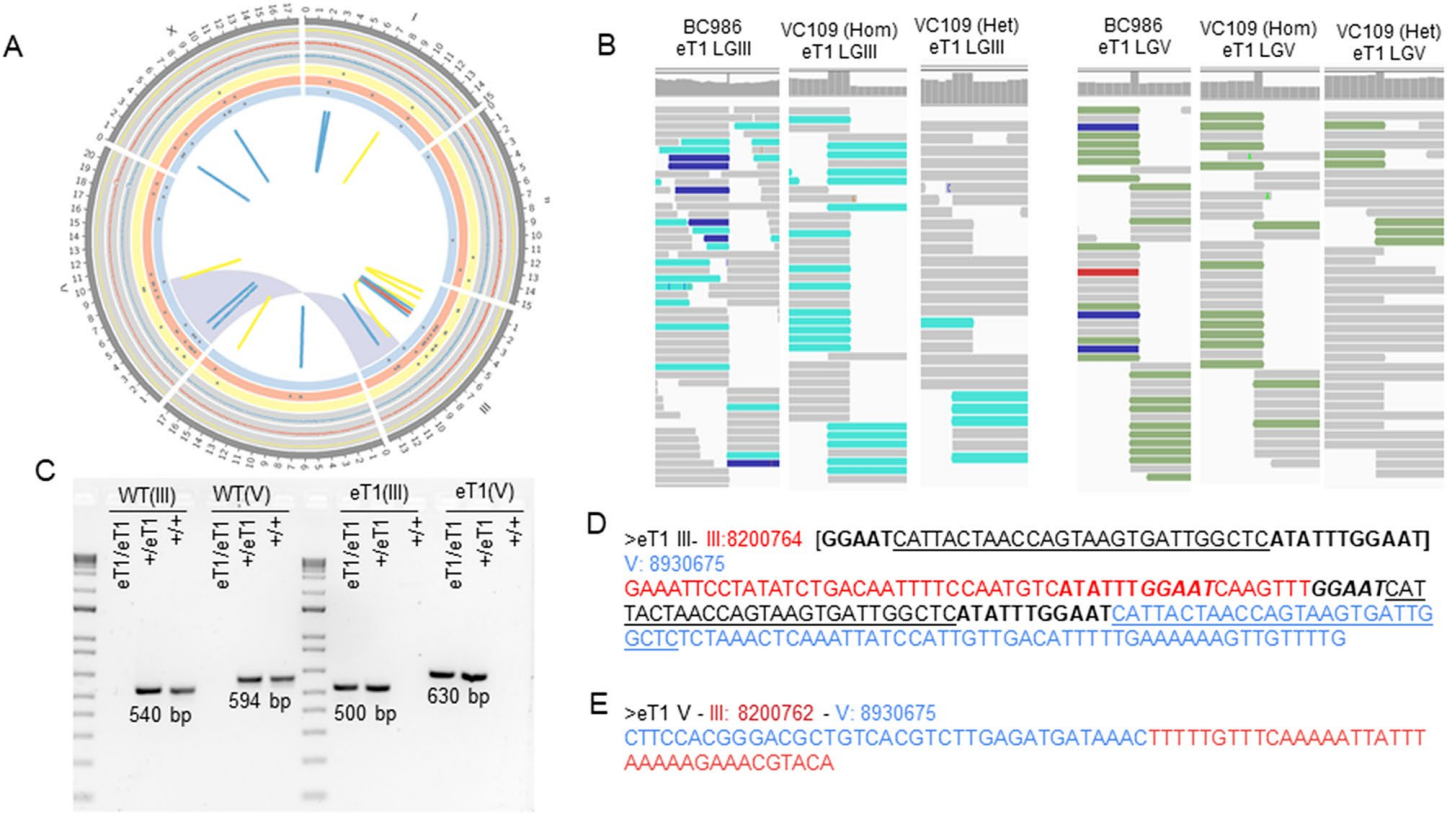
Overview of *eT1* genomes and validation of *eT1(III;V)* reciprocal translocation breakpoints. (**A**) Circos plot created with Circos^62^ with information regarding BC986 (yellow), wild-type looking VC109 (red; VC109 Het), and *unc-36* VC109 (blue; VC109 Hom) genomes. The outer section is composed of three-line charts (one per strain) representing the ratio of coverage calculated by windows of 1 Kb and divided by the strain-specific genome coverage. The middle section is composed of three scatter plots. Each dot represents the genomic position of heterozygous SNPs identified in each strain. The inner section highlights, with links and ribbons, SVs and complex rearrangements identified in each strain. The *eT1(III;V)* reciprocal translocation is displayed by a blue ribbon. For better resolution, see Supplemental Fig. S1. (**B**) Signature of reads aligned around the *eT1* breakpoints, visualized with IGV, BC986, wild-type looking VC109, and *unc-36* VC109 genomes. (**C**) PCR agarose gels validating *eT1* breakpoints and heterozygous and homozygous genotypes in VC109. (**D**, **E**) Sequences at *eT1* junctions on LGIII (**D**) and LGV (**E**) resolved by Sanger Sequencing. The junction on LGIII shows microhomologies with flaking regions. The sequence in red is from LGIII, the sequence in blue is from LGV. The sequence in black is the de novo sequence inserted at the junction, composed of microhomologies of the surrounding sequences. We represented the microhomologies between the junction sequence and the surrounding sequences with bold, italic and underlined characters.

Reads were aligned to the *C. elegans* reference genome (WS265) and candidate breakpoints were predicted using an ensemble of tools (see “Methods”). Two sets of breakpoints related to a translocation between LGIII and LGV were correctly identified by several tools in these *eT1* strains, but not in controls. The breakpoints we identified agreed with the locations previously described by Zhao and colleagues^32^: III:8,200,762–V:8,930,675 and III:8,200,764–V:8,930,675 (Fig. 1A and Supplemental Fig. S1). As a first validation step, we used the Integrative Genomics Viewer (IGV) to review the visual signature of reads aligned around those locations (Fig. 1B). In homozygous genomes, we observed that no read was overlapping the position of the breakpoint (i.e., the reads mostly aligned either on the left or the right of the breakpoint, with little or no read sequence aligning across the breakpoint position). In heterozygous genomes, half of the reads were displaying this signature (Fig. 1B). Then, we amplified the genomic loci around those breakpoints by PCR and submitted the PCR products for Sanger sequencing (Fig. 1C–E). By analyzing the Sanger sequences, we confirmed that the breakpoint on LGIII was in the second intron of *unc-36* at 8,200,764 Mb and that the breakpoint on LGV was intergenic, localized at 8,930,675 Mb. Additionally, we characterized microhomologies at the breakpoint on LGIII, composing a 43-bp sequence inserted at the junction containing several sequences flanking the breakpoints. The main part of the inserted sequence (27 bp) has been duplicated from the LGV flanking region. Two additional sequences, respectively 5 bp and 1 bp long, are duplicated from the LGIII flanking region (Fig. 1D).

One of the strains (VC109) was viable in both heterozygous and homozygous states^33^. We prepared genomic DNA from both *eT1* heterozygous (wild-type looking worms) and *eT1* homozygous (phenotypically *unc-36* worms) and sequenced them. We were able to identify (Fig. 1B) and confirm the *eT1* breakpoints in both cases (Fig. 1C), demonstrating that the short-read WGS approach is effective at deciphering position and structure of the breakpoints for reciprocal translocations regardless of the zygosity status.

### Short-read WGS contains enough information to identify short and large copy number variations

By combining calls from various tools, coverage analysis, and read inspection, we detected an assorted set of copy number variations. We confirmed their nature and positions by PCR and Sanger sequencing. Overall, we observed five deletions specific to BC986, spanning from 69 bp to 8,779 bp (Supplemental Figs. S2, S3). In VC109 genomes, we also detected four additional deletions ranging from 86 to 255 bp in size. Some were heterozygous, others were homozygous (Supplemental Figs. S4, S5, S6). We also identified two direct tandem copy number gain events in VC109. The first one, localized on LGI, was a homozygous direct tandem duplication in both heterozygous (phenotypically wild-type) and homozygous (phenotypically *unc-36*) worms. The second direct tandem duplication mapped on LGV and was both heterozygous and homozygous in heterozygous and homozygous worms, respectively (Supplemental Fig. S6). More information regarding these reported CNVs is available in Supplemental Table S1.

### Short-read WGS can uncover a free duplication

**Figure 2:**
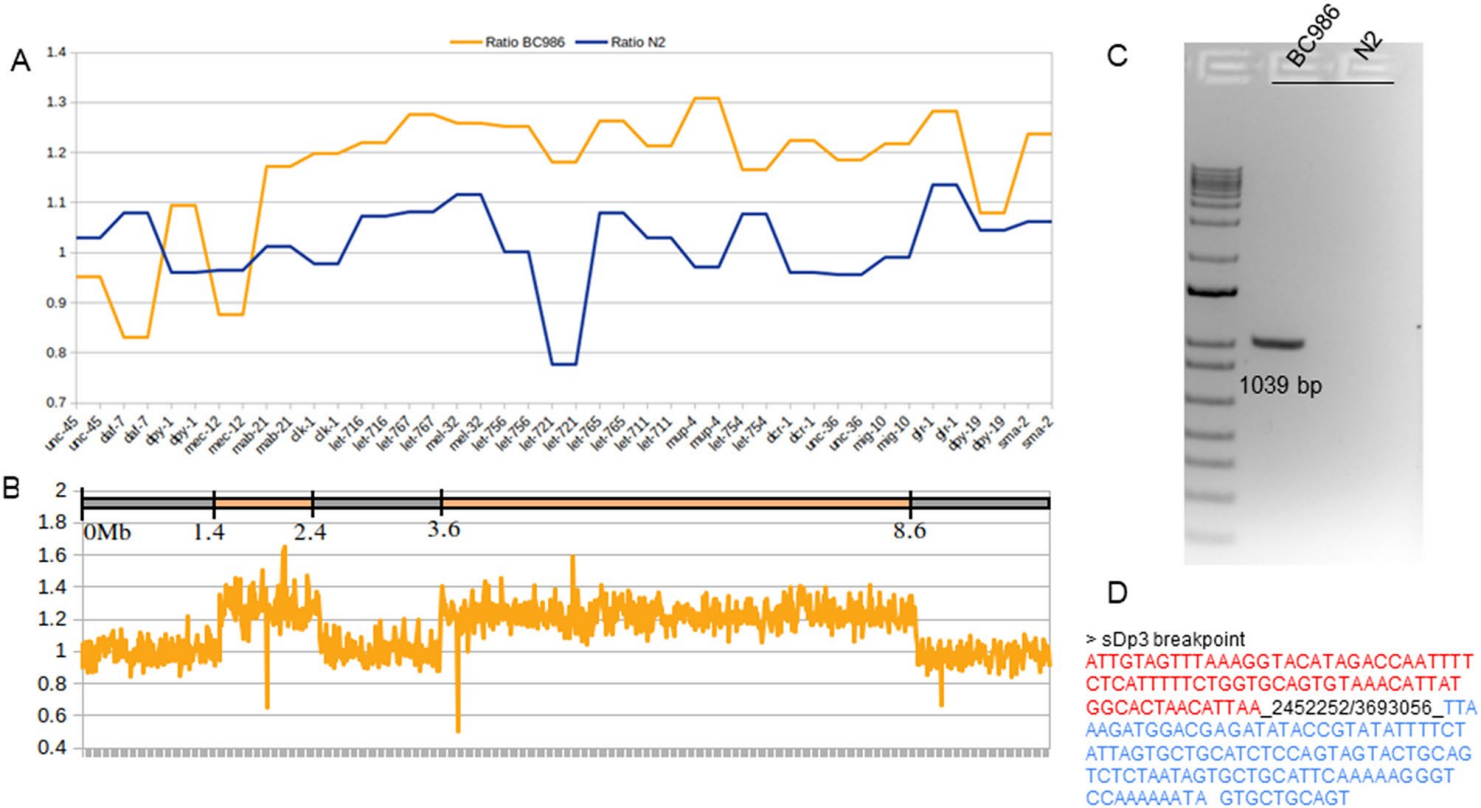
Free duplication *sDp3* in BC986. (**A**) Ratio of coverage for the 22 genes overlapped by the free duplication *sDp3* in BC986 (orange) and N2 (blue). (**B**) Coverage of LGIII in BC986 and *sDp3* boundaries. The map on the top displays the breakpoints identified for *sDp3* along LGIII. The line chart represents the ratio of coverage on the entire LGIII. The coverage was evaluated by a sliding window of 10 kb and divided by the average coverage for the entire genome. This ratio was then divided by the ratio of coverage for the same window in N2. (**C**) PCR gel confirming the breakpoints III:2,452,252 and III:3,693,056 linking the two duplicated segments of LGIII. (**D**) Sequence of the PCR product obtained by Sanger sequencing. The red sequence belongs to the first part of the free duplication (III:1.4–2.4 Mb) and the blue sequence belongs to the second portion of the duplication (III3.6–8.6 Mb).

The *sDp3* balancer, also present in BC986 along with *eT1(III;V),* has been described as a free duplication on LGIII effectively balancing the left portion of LGIII from around *unc-86* through to at least *dpy-1*, but does not extend to *unc-45*^28^. So far, 22 genes have been described to be overlapped by *sDp3* and, by analysis of the coverage, we confirmed that their sequences were duplicated (Fig. 2A). None of the tools we applied (see “Methods”) reported breakpoints or structural variants that could fit the *sDp3* description. However, we observed heterozygous SNVs from the left end of LGIII until at least the *eT1* breakpoint (III:8,200,675), corroborating the presence of an event balancing this part of LGIII, and maintaining heterozygosity (Supplemental Fig. S2 and Supplemental Table S3). An unbiased analysis of the sequencing read depth on LGIII helped us map the duplication to two different loci: between III:1.4 Mb-2.4 Mb and III:3.6 Mb-8.6 Mb (Fig. 2B). To confirm this structure, we inspected the reads aligned around III:2.4 Mb and III:3.6 Mb. We identified read pairs for which the forward read was aligned to the first segment of the duplication and the mate aligned along the second segment, thus corroborating our hypothesis. To experimentally validate it, we identified the breakpoint linking the two parts of the duplication (III:2,452,252 and III:3,693,056) and confirmed by PCR and Sanger sequencing (Fig. 2C and D).

### Short-read WGS can reveal and characterize unexpected complex rearrangements

By comparing variants and breakpoints in the three *eT1* strains and controls—strains without *eT1(III;V)* including N2 and BC4586, we built an “*eT1* haplotype” composed of variants specific to the *eT1* strains. Interestingly, along with eight SNVs (list available in Supplemental Table S2), we also characterized two unexpected and undescribed complex rearrangements.

The first one could have been interpreted at first sight as a classic large copy number gain in tandem (direct) spanning from V:2,144,217 to V:2,156,311 (Supplemental Fig.S7A). It overlapped seven intact genes: *srbc-20*, *C45H4.t1*, *C45H4.21*, *C45H4.13*, *C45H4.19*, *srbc-24* and, *srbc-23*, as well as partially spanning *srbc-52* (exon 1 only) and *srbc-21* (up to intron 4). PCR and Sanger sequencing confirmed the duplication breakpoints and structure in direct tandem (Supplemental Fig. S7B). Both BC986 and *unc-36* VC109 worms [*eT1(III;V)* homozygous] were homozygous for the direct tandem duplication (ratio of coverage = 2) while wild-type looking VC109 [*eT1(III;V)* heterozygous] was heterozygous (ratio of coverage = 1.5) (Supplemental Fig. S7C). The analysis of the coverage however showed a discontinuity in the coverage (ratio of coverage dropping back to 1) between V:2,148,200 and V:2,148,630 which corresponds to the three last exons of *srbc-20* and a part of the last exon of *C45H4.21* (Supplemental Fig. S7C). An inspection of the reads revealed that this variant is complex, with an inversion overlapping the copy number gain. We confirmed the inversion V:2,148,056–2,148,630 by PCR and Sanger sequencing (Supplemental Fig. S7B and D).

**Figure 3:**
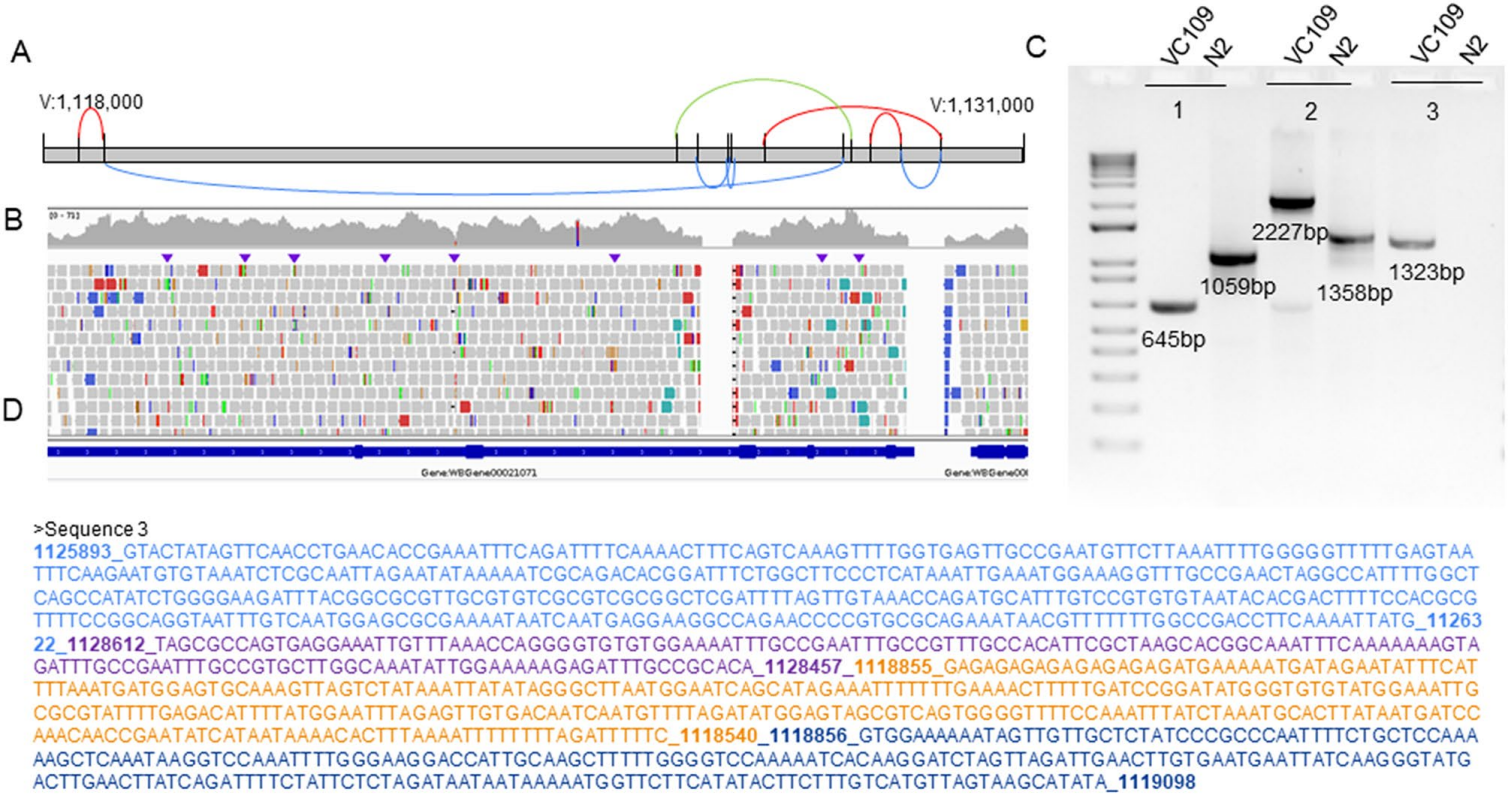
Complex rearrangement in *eT1* strains on LGV. (**A**) Schematic representation of the complex rearrangement combining three inverted tandem duplications (red), four deletions (blue), and one inversion (green) along LGV around 1.1 Mb. (**B**) IGV screenshot of the VC109 genome (*unc-36*) for the region overlapped by the complex rearrangements. (**C**) PCR gels. 1 = Deletions V:1,126,582–1,126,983 and V:1,127,007–1,127,020; 2 = Deletion V:1,129,264–1,129,753, inverted tandem duplication V:1,127,471–1,129,752 and inverted tandem duplication V:1,128,827–1,129,264. 3 = Deletion V:1,118,855–1,128,457, inverted tandem duplication V:1,118,539–1,118,853, and inversion V:1,126,322–1,128,612. (**D**) Sanger sequencing of the PCR product #3 confirming several breakpoints.

The second *eT1* specific complex rearrangement was localized on LGV around 1.1 Mb. The complex rearrangement described here overlapped with the gene *Y50D4B.1*, a non-essential gene in *C. elegans*. Between V:1.118 Mb and V:1.130 Mb, we identified 15 different breakpoints (Table 1). By inspecting the reads, we identified three short deletions (homozygous in BC986 and VC109 *unc-36* worms, heterozygous in VC109 wild-type worms), one inversion, one large deletion and three inverted tandem duplications (Fig. 3). We confirmed experimentally all breakpoints by PCR and Sanger sequencing.

**Table 1:** Breakpoints of the complex rearrangement present in *eT1* containing strains on LGV.

Variant	Breakpoint 1	Breakpoint 2	Gene
Inverted tandem duplication	V:1,118,539	V:1,118,853	*Y50D4B.1*
Deletion	V:1,118,855	V:1,128,457	*Y50D4B.1*
Deletion	V:1,126,582	V:1,126,983	*Y50D4B.1*
Deletion	V:1,127,007	V:1,127,020	*Y50D4B.1*
Inverted tandem duplication	V:1,127,471	V:1,129,752	*Y50D4B.1*
Inverted tandem duplication	V:1,128,827	V:1,129,264	*Y50D4B.1*
Deletion	V:1,129,264	V:1,129,753	*Y50D4B.1*
Inversion	V:1,126,322	V:1,128,612	*Y50D4B.1*

**Figure 4:**
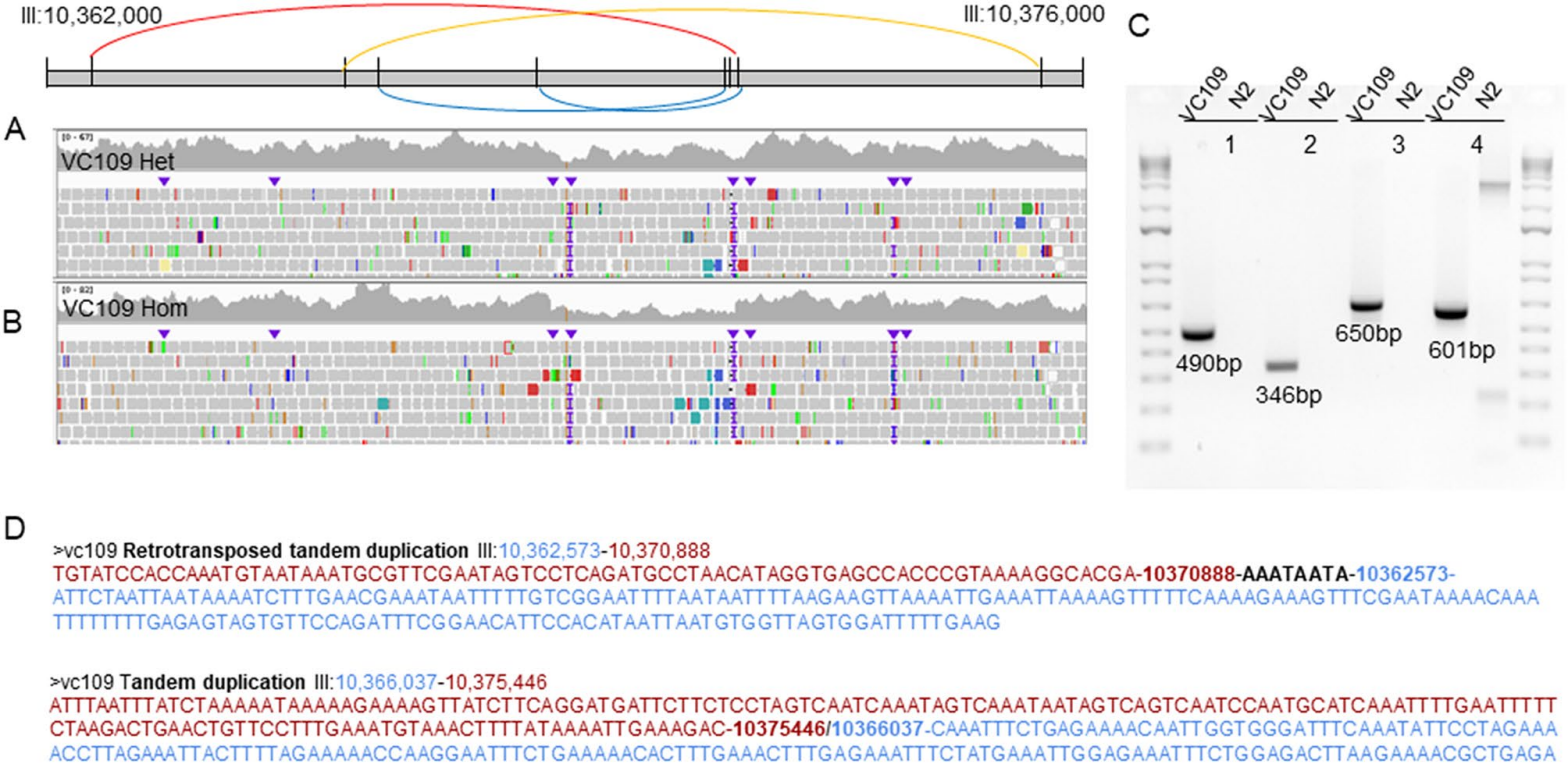
Complex rearrangement in VC109 on LGIII. (**A**) Schematic representation of the complex rearrangement combining one inverted tandem duplication (red), one direct tandem duplication (orange), and two deletions (blue) along LGIII around 10 Mb. (**B**) IGV screenshot for the region overlapped by the complex rearrangements. (**C**) PCR gels. 1 = Inverted tandem duplication III:10,362,573–10,370,888; 2 = Deletion III:10,366,492–10,370,879; 3 = Direct tandem duplication III:10,366,037–10,375,446; 4 = Deletion III:10,368,666–10,371,166. (**D**) Sequences obtained with Sanger sequencing displaying regions surrounding breakpoint junctions for the two duplications of the complex rearrangement. The blue and red sequences represent the sequence surrounding the breakpoints and coming from parts of the reference genome away from each other.

In the strain VC109 only, we detected eight breakpoints on LGIII around 10 Mb (Table 2 and Fig. 4). Based on coverage analysis and PCR, we further characterized this complex rearrangement as being composed of one direct tandem duplication, one inverted tandem duplication, and two deletions. Because of the presence of copy gains in the rearrangement and microhomologies at breakpoint junctions, this complex rearrangement could be characterized as chromoanasynthesis. It overlapped the non-essential gene *tbc-8*, so it is not expected to have an important effect on the fitness of the worms.

**Table 2:** Complex rearrangement breakpoints in VC109 on LGIII.

Variant	Length	Breakpoint 1	Breakpoint 2	Gene
Inverted tandem duplication	8315 bp	III:10,362,573	III:10,370,888	*tbc-8*
Deletion	4387 bp	III:10,366,492	III:10,370,879	*tbc-8*
Direct tandem duplication	9409 bp	III:10,366,037	III:10,375,446	*tbc-8*
Deletion	2500 bp	III:10,368,666	III:10,371,166	*tbc-8*

### Short-read WGS to characterize BC4586, an uncharacterized genetic balancer strain

The strain BC4586 contains the *sC4* rearrangement that has been used to balance the right end of LGV, from *rol-9* to *unc-76*. It was also reported that it reduces the genetic distance between the genes *unc-76* and *rol-9* to 1.8%, suggesting the presence of a deletion^28^. To the best of our knowledge, the rearrangement *sC4* remains molecularly uncharacterized. We used short-read WGS to determine the nature of the *sC4* rearrangement and to report additional genomic variants in BC4586.

**Figure 5:**
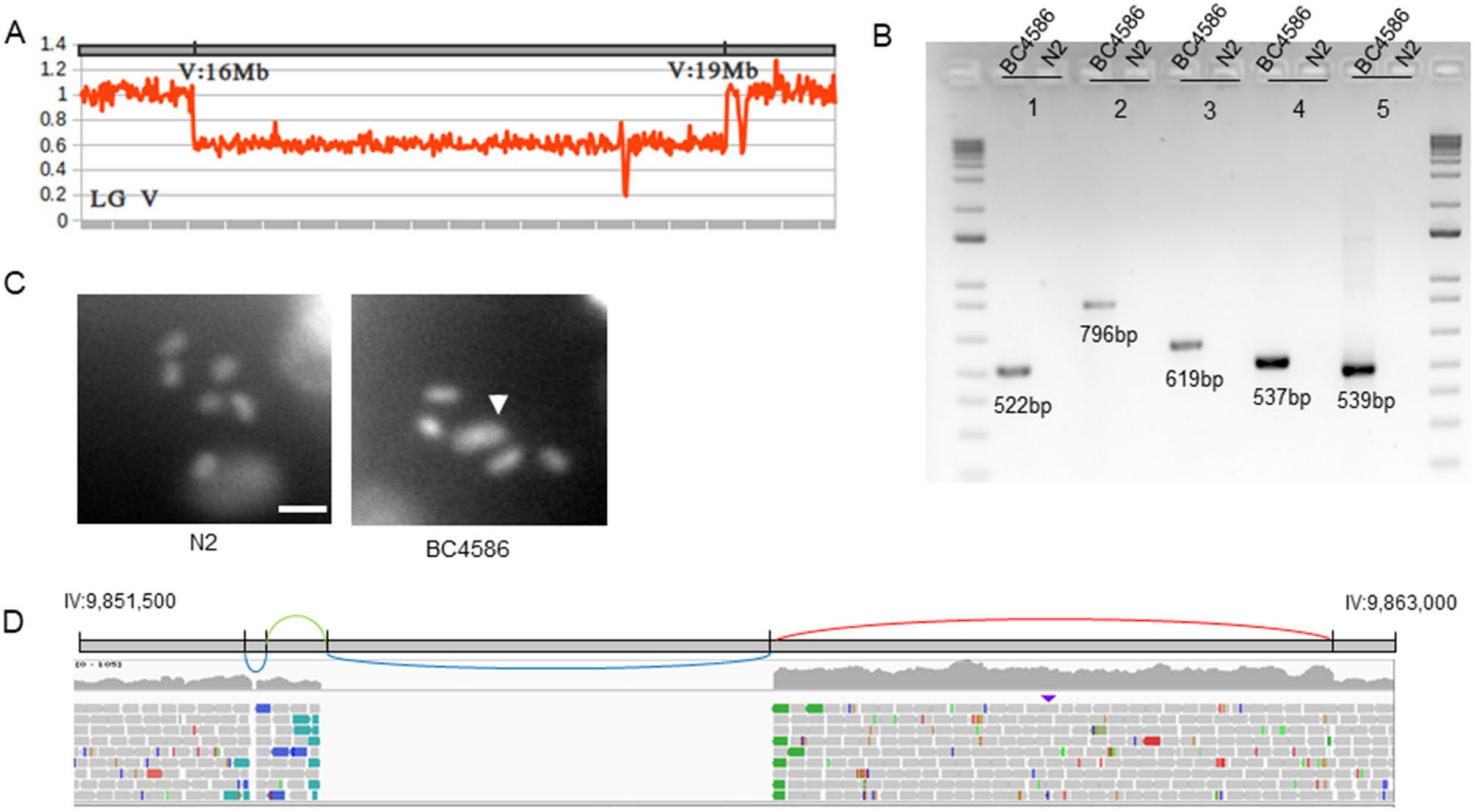
Characterization of the balancer *sC4* and a new complex rearrangement in BC4586 on LGIV. (**A**) Coverage analysis of the region surrounding the deletion of the segment V:16,060,619–19,331,432 in BC4586, revealing a large deletion. (**B**) PCR gels validating SVs and complex rearrangements in BC4586. 1 = Direct tandem duplication III:5,059,444–5,063,035; 2 = Deletion IV:9,853,074–9,853,123, inversion IV:9,853,123–9,853,675 and deletion IV:9,853,675–9,857,585; 3 = Direct tandem duplication IV:9,857,585–9,862,397; 4 = Deletion *sC4* V:16,060,619–19,331,432; 5 = Deletion V:20,780,774–20,781,638. The breakpoints validated with the PCR no2 and no3 are part of the same chromosomal rearrangement. (**C**) Representative karyotypes for N2 and BC4586, confirming *sC4* chromosome fusion (IV;V) (indicated by a white arrow) in BC4586. The scale bar represents 4 uM. (**D**) Schematic representation of the chromosomal rearrangement on LGIV. The successive SVs are displayed along the genomic region IV:9,851,500–9,863,000. The chromosomal rearrangement is composed of two deletions (blue), one direct tandem duplication (red), and one inversion (green). IGV screenshot of BC4586 aligned reads along the genomic region IV:9,851,500–9,863,000.

We first performed “*sC4* haplotype” analysis (Supplemental Table S3) and observed stretches of heterozygous SNVs only on LGV from ~ 12 to ~ 16 Mb and from ~ 19 Mb to its right end. This suggested that *sC4* might be able to balance further than *unc-76*. We detected a deletion on the right portion of LGV between 16 and 19 Mb (Fig. 5A) that explains the reduced genetic distance previously reported between *unc-76* and *rol-9*. We confirmed the deletion by PCR (Fig. 5B). We have also detected a non-reciprocal translocation of the right arm of LGV to the right arm of LGIV (Table 3). We hypothesized that this has led to a fusion of the two chromosomes, by their right ends. The breakpoint was supported by several reads. However, the region surrounding the breakpoint on LGV is highly repetitive, and despite our best efforts, we could not design a unique set of primers to validate this hypothesis by Sanger. Therefore, we assessed the karyotypes of the diakinetic oocytes using DAPI staining. The wild-type oocytes typically have six pairs of DAPI-stained bivalent diakinetic chromosomes (Fig. 5C), whereas in the BC4586, we frequently observe five pairs (Fig. 5C), confirming *sC4* chromosome fusion.

**Table 3:** SVs and complex rearrangements in BC4586.

Variant	Breakpoint 1	Breakpoint 2	Overlapped genes
Deletion	V:16,060,619	V:19,331,432	1279 genes
Translocation	IV:17,114,723	V:19,835,910	*cyn-13*
Deletion	IV:9,853,074	IV:9,853,123	*ssq-1; K07F5.12* ; *npp-1*
Inversion	IV:9,853,123	IV:9,853,675	
Deletion	IV:9,853,675	IV:9,857,585	
Direct tandem duplication	IV:9,857,585	IV:9,862,397	

On LGIV, we also characterized a complex homozygous rearrangement combining two deletions, one inversion and one direct tandem duplication localized around 9.8 Mb (Fig. 5D). We confirmed the breakpoints for both complex rearrangements by PCR (Fig. 5D). We also reported and validated a deletion on LGV and a direct tandem duplication on LGIII (Fig. 5D, Supplemental Table S1, Supplemental Fig. S8). The Circos plot in Supplemental Fig. S9 summarizes our findings.

## Discussion

Short-read whole genome sequencing (WGS) has often been used to retrieve structural variants and more complex rearrangements among other variations in humans^17,34,35^, *D. melanogaster*^36^, as well as *C. elegans*^37–40^. Here, by reporting the precise breakpoints of complex rearrangements in *C. elegans* [*eT1(III;V), sC4, sDp3*], we describe the molecular structure of widely used balancers, most of them for the first time to the best of our knowledge. We also show that short-read WGS enables identification and characterization of large SVs and complex rearrangements, by deep analysis of short-read WGS datasets.

Every breakpoint that we uncovered with deep analysis of the short reads was validated experimentally. However, the interpretation of the structure of the rearrangements could necessitate further exploration. Still, despite the limited ability of short-read WGS to span large genomic rearrangements fully or to explore repetitive regions such as telomeres, we characterized the balancer *sC4* as a large deletion and a chromosome fusion (*IV;V*). This rearrangement could reflect a telomere crisis^41^ occurring as an end-to-end chromosome fusion associated with telomere shortening. This type of event has been previously studied in *C. elegans*^38,42,43^. We also uncovered a free duplication, composed of two genomic segments (*sDp3*), along with chromosomal rearrangements combining several various events that present features of chromoanagenesis. Our analyses of *eT1* strains confirmed the *eT1* breakpoints as identified by Zhao and colleagues^32^. Interestingly, we placed the LGIII breakpoint 3-bp anterior to the one previously reported. Although we retrieved the junction sequence described as a 35-bp duplication, our approach with short-read WGS showed a more complex scenario with microhomologies of several flanking sequences, suggesting the involvement of a replication-based DNA mechanism repair such as fork-stalling and template switching, or microhomology-mediated break-induced replication.

In *C. elegans*, short-read WGS has been employed in only a few studies to describe SVs. For instance, Meier and colleagues^38^ and Volkova and colleagues^39^ reported mutational signatures (SNVs and SVs) created by carcinogen exposure on strains with DNA repair deficiency. Itani and colleagues^37^ characterized a complex rearrangement created by ENU-based mutagenesis. In 2017, Cook and colleagues^44^ published the database CeNDR (*C. elegans* Natural Diversity Resource) that regroups genomic variations uncovered by genome sequencing in wild *C. elegans* strains. Other than insertions of transposable elements^45^, SVs and complex rearrangements are not reported for the natural isolates in the CeNDR. Our study shows that short-read sequencing is a viable option for future studies to explore the natural variation of *C. elegans* species beyond SNVs, especially by re-analyzing datasets already available in CeNDR, for which those complex variants might have been overlooked.

It is quite common in human studies to assess analysis pipelines of short-read WGS using either generic genomes (Genome in a Bottle) or simulated data^46,47^, especially because real-life cases emerge anecdotally. However, in our study, we assessed several tools and approaches on real biological data from model organism genomes. This approach presents two main advantages. First, in model organisms like *C. elegans*, balancers are widely used and well-known as being genomes containing SVs and complex rearrangements, largely comparable to humans. Thus, they constitute good surrogates of real-life cases, without the limitation related to a low frequency of those events. Second, as shown here, real biological data allows us to uncover unexpected events, of various natures and complexity. Thus, there is a probability that simulations might not be able to cover the wide diversity of chromosomal rearrangements or might not simulate the complexity of read signatures. Thus, we reasoned that tool assessment would be more accurate if they were performed on real data, human or not, combined with experimental validation.

## Conclusions

In our study, we showed that short-read data provides enough information to detect a spectrum of complex variants with tailored bioinformatics approaches. Thus, to improve the detection and characterization of SVs and complex rearrangements, it is important to also optimize pipelines and analyses to get the best out of the short-read datasets. Indeed, short-read sequencing is the most widely used approach and the most cost-effective technology available. Also, as there are more tools and pipelines available to analyze short-read data than for long-read or linked-read data, it facilitates pipeline tailoring by using different tools and approaches. Additionally, short-reads permit the detection of both single nucleotide variants and larger ones, whereas long-read approaches are error prone and thus, limited, in their ability to accurately detect SNVs. This constitutes quite an advantage for short-read approach as it avoids the necessity to resort to another assay for small variants. Moreover, public databases on human variation such as TopMed^48^ and gnomAD^49^ have been built upon calls from short-read datasets. Therefore, in the context of human rare disease unsolved cases, where population databases are a major asset to decipher rare and pathogenic variants from common and benign ones, short-read sequencing remains the main approach. Thus, improving short-read sequencing pipelines to maximize the detection of variants is of utmost importance.

## Methods

### Worm maintenance and strains

Strains Bristol N2 wild type, Hawaiian strain wild type CB4856, BC986 *(sDp3(III;f);* + */eT1 (III;V))*, VC109 *(apc-11(gk37)/eT1 III;* + */eT1 V)* and BC4586 *(unc-76(e911) rol-9(sc148)/sC4(s2172) [dpy-21(e428)] V)* were used in this study. Strains were obtained from the CGC (Caenorhabditis Genetics Center). N2 was used as the wildtype strain. All strains were maintained at 16 °C and kept on standard NGM plates streaked with OP50.

### DNA extraction

Genomic DNA was collected from approximately 100 mg of worm tissue using the Qiagen Blood and Tissue kit (Cat #: 13323) following the manufacturer's recommendations. DNA was eluted with 10 mM Tris–HCl (pH 8.0). Samples were quality-checked to ensure a minimum quantity of 1500 ng and a 260/280 ratio of 1.8 before submitting for sequencing.

### Library preparation, sequencing and data pre-processing

Paired-end short-read WGS were obtained for all strains with PCR-free library preparation protocol and NovaSeq6000 Illumina sequencing technology. We checked the quality of the fastq files using FastQC^50^. The reads were 151 bp long. We trimmed the reads and removed the adapters using TRIMMOMATIC v0.36^51^. For each sample, we aligned between 16 and 34 million reads using BWA-MEM v0.7.17^52^ algorithm to the *C. elegans* reference genome WS265. It resulted in a 30X read coverage per strain on average (Supplemental Table S4). We then sorted the reads according to their coordinates with ‘samtools sort’ (samtools v1.5)^53^.

### SV and complex rearrangement detection

We called and characterized SNVs, indels, SVs, and complex rearrangements for each strain in this study using a collection of published tools and downstream in-house designed analysis methods. Strain N2 was used as a control. The SNVs and indels genotype of each strain was established using RUFUS^35^. The analysis of SNV heterozygosity along the genome of each strain was used to highlighted balanced genomic regions. For SVs and complex variants, we initially ran nine different tools with default parameters: BreakDancer v.BreakDancerMax-1.1r112^54^ (https://github.com/genome/breakdancer), CNVnator v0.4.1^55^ (https://github.com/abyzovlab/CNVnator), DELLY v0.7.8^56^ (https://github.com/dellytools/delly), GRIDSS v2.8.0^57^ (https://github.com/PapenfussLab/GRIDSS), Manta v1.6.0^58^ (https://github.com/Illumina/manta), SeekSV v1.2.3^59^ (https://github.com/qiukunlong/seeksv), Tardis v1.0.7^60^ (https://github.com/BilkentCompGen/tardis), TIDDIT v2.12.0^61^ (https://github.com/SciLifeLab/TIDDIT) and RUFUS^35^ (https://github.com/jandrewrfarrell/RUFUS). For complex variants, breakpoints were defined combining RUFUS and GRIDSS calls and custom methods (visual assessment, coverage analysis, reads inspection):

1. Visual assessment consists in reviewing the visual signature of reads aligned around each breakpoint with IGV. A breakpoint is represented by accumulation of split reads, with little or no read sequence aligning across the breakpoint position. The visual signature gives information to characterize the type of rearrangement^29^.
2. Read inspection consists in a “manual” re-alignment of reads aligned at each breakpoint junction. Reads are extracted from bam files with “samtools view” and re-aligned using Blast (UCSC – Feb. 2013; WBcel235/ce11). This analysis aims to identify split reads supporting the breakpoint junctions (as described by Iwata et al.^29^). Such reads are fundamental to the design of PCR primers for further validation.
3. To characterize copy number variations (stand-alone CNVs or as part of complex variants), we estimated the average genome coverage and read depth by intervals of 1–10 kb (depending on the length of the CNV) using the ‘samtools depth’ function.

The circular visualizations were produced using Circos^62^. The line charts were prepared in Excel. The data relating to the genomic variations are available in Supplementary information for BC4586 (Supplemental Table S5) and VC109 (Supplemental Tables S6 and S7) genomes. The complete list of additional SVs identified and confirmed by PCR, but not discussed in this paper, is available in Supplemental Table S1. Circos plots, PCR gels, and IGV screenshots are available in Supplemental Figures S1, S2, S3, S4, S5, S6, S7, S8, S9.

### Experimental validation

We confirmed breakpoints of SVs and complex rearrangements by PCR and Sanger Sequencing. All primers and sequences are available in Supplementary Information (Supplemental Tables S8, S9, S10, S11). For the cytological assessment of bivalent diakinetic oocyte karyotypes, 1-day-old adult hermaphrodite worms were washed once in M9 medium, fixed in cold methanol, rehydrated in PBS (0.01% Tween) and mounted using SlowFade Gold antifade reagent with DAPI (Invitrogen S36938). Images were acquired using a Zeiss Imager M2. Raw counts can be found in Supplemental Table S12. The *p-value* was calculated using two-tailed Z-test.

## Supplementary Information

Supplementary Figure 1.

Supplementary Figure 2.

Supplementary Figure 3.

Supplementary Figure 4.

Supplementary Figure 5.

Supplementary Figure 6.

Supplementary Figure 7.

Supplementary Figure 8.

Supplementary Figure 9.

Supplementary Information.

Supplementary Tables.

## Data Availability

The sequencing data generated in this study have been submitted to the NCBI BioProject database (https://www.ncbi.nlm.nih.gov/bioproject/) under accession number PRJNA728090.

## References

[1] Pellestor F, Gaillard J, Schneider A, Puechberty J, Gatinois V (2021). Chromoanagenesis, the mechanisms of a genomic chaos. Semin. Cell Dev. Biol..

[2] Cortés-Ciriano I (2020). Comprehensive analysis of chromothripsis in 2,658 human cancers using whole-genome sequencing. Nat. Genet..

[3] Goldrich DY (2021). Identification of somatic structural variants in solid tumors by optical genome mapping. J. Pers. Med..

[4] Tommerup N (1993). Mendelian cytogenetics. Chromosome rearrangements associated with mendelian disorders. J. Med. Genet..

[5] Kloosterman WP (2011). Chromothripsis as a mechanism driving complex de novo structural rearrangements in the germline. Hum. Mol. Genet..

[6] Maroilley T, Tarailo-Graovac M (2019). Uncovering missing heritability in rare diseases. Genes.

[7] Zepeda-Mendoza CJ, Morton CC (2019). The iceberg under water: Unexplored complexity of chromoanagenesis in congenital disorders. Am. J. Hum. Genet..

[8] Anzick S (2020). Chromoanasynthesis as a cause of Jacobsen syndrome. Am. J. Med. Genet. A.

[9] Arya P, Hodge JC, Matlock PA, Vance GH, Breman AM (2021). Two patients with complex rearrangements suggestive of germline chromoanagenesis. Cytogenet. Genome Res..

[10] Belyeu JR (2021). De novo structural mutation rates and gamete-of-origin biases revealed through genome sequencing of 2396 families. Am. J. Hum. Genet..

[11] Du H (2021). Analysis of structural variants reveal novel selective regions in the genome of Meishan pigs by whole genome sequencing. Front. Genet..

[12] Langner T (2021). Genomic rearrangements generate hypervariable mini-chromosomes in host-specific isolates of the blast fungus. PLoS Genet..

[13] Crow T (2020). Gene regulatory effects of a large chromosomal inversion in highland maize. PLoS Genet..

[14] Zhao Y (2020). A spontaneous complex structural variant in rcan-1 increases exploratory behavior and laboratory fitness of *Caenorhabditis elegans*. PLoS Genet..

[15] Begum G (2021). Long-read sequencing improves the detection of structural variations impacting complex non-coding elements of the genome. Int. J. Mol. Sci..

[16] Liu Y (2020). Comparison of multiple algorithms to reliably detect structural variants in pears. BMC Genom..

[17] Neerman N (2019). A clinically validated whole genome pipeline for structural variant detection and analysis. BMC Genom..

[18] Cameron DL, Di Stefano L, Papenfuss AT (2019). Comprehensive evaluation and characterisation of short read general-purpose structural variant calling software. Nat. Commun..

[19] Kosugi S (2019). Comprehensive evaluation of structural variation detection algorithms for whole genome sequencing. Genome Biol..

[20] Uguen K (2020). Genome sequencing in cytogenetics: Comparison of short-read and linked-read approaches for germline structural variant detection and characterization. Mol. Genet. Genomic Med..

[21] Onishi-Seebacher M, Korbel JO (2011). Challenges in studying genomic structural variant formation mechanisms: The short-read dilemma and beyond. BioEssays News Rev. Mol. Cell. Dev. Biol..

[22] Yang L (2020). A practical guide for structural variation detection in the human genome. Curr. Protoc. Hum. Genet..

[23] Ebert P (2021). Haplotype-resolved diverse human genomes and integrated analysis of structural variation. Science.

[24] Mizuguchi T (2019). A 12-kb structural variation in progressive myoclonic epilepsy was newly identified by long-read whole-genome sequencing. J. Hum. Genet..

[25] Thibodeau ML (2020). Improved structural variant interpretation for hereditary cancer susceptibility using long-read sequencing. Genet. Med. Off. J. Am. Coll. Med. Genet..

[26] Lei M (2020). Long-read DNA sequencing fully characterized chromothripsis in a patient with Langer-Giedion syndrome and Cornelia de Lange syndrome-4. J. Hum. Genet..

[27] Merker JD (2018). Long-read genome sequencing identifies causal structural variation in a Mendelian disease. Genet. Med. Off. J. Am. Coll. Med. Genet..

[28] Edgley ML, Baillie DL, Riddle DL, Rose AM (2006). Genetic balancers. WormBook Online Rev. C Elegans Biol..

[29] Iwata S, Yoshina S, Suehiro Y, Hori S, Mitani S (2016). Engineering new balancer chromosomes in *C. elegans* via CRISPR/Cas9. Sci. Rep..

[30] Dejima K (2018). An aneuploidy-free and structurally defined balancer chromosome toolkit for *Caenorhabditis elegans*. Cell Rep..

[31] Rosenbluth RE, Baillie DL (1981). The genetic analysis of a reciprocal translocation, eT1(III; V), in *Caenorhabditis elegans*. Genetics.

[32] Zhao Y (2006). A mutational analysis of *Caenorhabditis elegans* in space. Mutat. Res..

[33] C. elegans Deletion Mutant Consortium. Large-scale screening for targeted knockouts in the *Caenorhabditis elegans* genome. *G3 Bethesda Md* **2**, 1415–1425 (2012). 10.1534/g3.112.003830 PMC3484672 23173093

[34] Campbell PJ (2008). Identification of somatically acquired rearrangements in cancer using genome-wide massively parallel paired-end sequencing. Nat. Genet..

[35] Ostrander BEP (2018). Whole-genome analysis for effective clinical diagnosis and gene discovery in early infantile epileptic encephalopathy. Npj Genomic Med..

[36] Miller DE (2016). Whole-Genome analysis of individual meiotic events in drosophila melanogaster reveals that noncrossover gene conversions are insensitive to interference and the centromere effect. Genetics.

[37] Itani OA, Flibotte S, Dumas KJ, Moerman DG, Hu PJ (2015). Chromoanasynthetic genomic rearrangement identified in a n-ethyl-n-nitrosourea (ENU) mutagenesis screen in *Caenorhabditis elegans*. G3 Bethesda Md.

[38] Meier B (2014). *C. elegans* whole-genome sequencing reveals mutational signatures related to carcinogens and DNA repair deficiency. Genome Res..

[39] Volkova NV (2020). Mutational signatures are jointly shaped by DNA damage and repair. Nat. Commun..

[40] Hillier LW (2008). Whole-genome sequencing and variant discovery in *C. elegans*. Nat. Methods.

[41] McClintock B (1941). The stability of broken ends of chromosomes in Zea Mays. Genetics.

[42] Meier B, Volkova NV, Gerstung M, Gartner A (2020). Analysis of mutational signatures in *C. elegans*: Implications for cancer genome analysis. DNA Repair.

[43] Hillers KJ, Villeneuve AM (2003). Chromosome-wide control of meiotic crossing over in *C. elegans*. Curr. Biol. CB.

[44] Cook DE, Zdraljevic S, Roberts JP, Andersen EC (2017). CeNDR, the *Caenorhabditis elegans* natural diversity resource. Nucl. Acids Res..

[45] Laricchia KM, Zdraljevic S, Cook DE, Andersen EC (2017). Natural variation in the distribution and abundance of transposable elements across the *Caenorhabditis elegans* species. Mol. Biol. Evol..

[46] Li Z (2020). VarBen: Generating in silico reference data sets for clinical next-generation sequencing bioinformatics pipeline evaluation. J. Mol. Diagn. JMD.

[47] Richmond PA (2020). GeneBreaker: variant simulation to improve the diagnosis of Mendelian rare genetic diseases. Hum. Mutat..

[48] Burgess DJ (2021). The TOPMed genomic resource for human health. Nat. Rev. Genet..

[49] Karczewski KJ (2020). The mutational constraint spectrum quantified from variation in 141,456 humans. Nature.

[50] Andrews S. *FastQC: A Quality Control Tool for High Throughput Sequence Data*. (2010).

[51] Bolger AM, Lohse M, Usadel B (2014). Trimmomatic: A flexible trimmer for Illumina sequence data. Bioinforma. Oxf. Engl..

[52] Li, H. Aligning sequence reads, clone sequences and assembly contigs with BWA-MEM. http://arxiv.org/abs/13033997 Q-Bio (2013).

[53] Li H (2009). The sequence alignment/map format and SAMtools. Bioinforma. Oxf. Engl..

[54] Fan, X., Abbott, T. E., Larson, D. & Chen, K. BreakDancer: Identification of genomic structural variation from paired-end read mapping. *Curr. Protoc. Bioinforma.* **45**, 15.6.1–11 (2014). 10.1002/0471250953.bi1506s45 PMC4138716 25152801

[55] Abyzov A, Urban AE, Snyder M, Gerstein M (2011). CNVnator: An approach to discover, genotype, and characterize typical and atypical CNVs from family and population genome sequencing. Genome Res..

[56] Rausch T (2012). DELLY: structural variant discovery by integrated paired-end and split-read analysis. Bioinforma. Oxf. Engl..

[57] Cameron DL (2017). GRIDSS: sensitive and specific genomic rearrangement detection using positional de Bruijn graph assembly. Genome Res..

[58] Chen X (2016). Manta: rapid detection of structural variants and indels for germline and cancer sequencing applications. Bioinforma. Oxf. Engl..

[59] Liang Y (2017). Seeksv: an accurate tool for somatic structural variation and virus integration detection. Bioinforma. Oxf. Engl..

[60] Soylev A, Kockan C, Hormozdiari F, Alkan C (2017). Toolkit for automated and rapid discovery of structural variants. Methods San Diego Calif.

[61] Eisfeldt J, Vezzi F, Olason P, Nilsson D, Lindstrand A (2017). TIDDIT, an efficient and comprehensive structural variant caller for massive parallel sequencing data. F1000Research.

[62] Krzywinski M (2009). Circos: An information aesthetic for comparative genomics. Genome Res..

