# Supplementary figures and images for: Deciphering complex genome rearrangements in C. elegans using short-read whole genome sequencing
Source: Sci Rep. 2021 Sep 14;11:18258. doi: 10.1038/s41598-021-97764-9 (PMC8440550; doi:10.1038/s41598-021-97764-9)

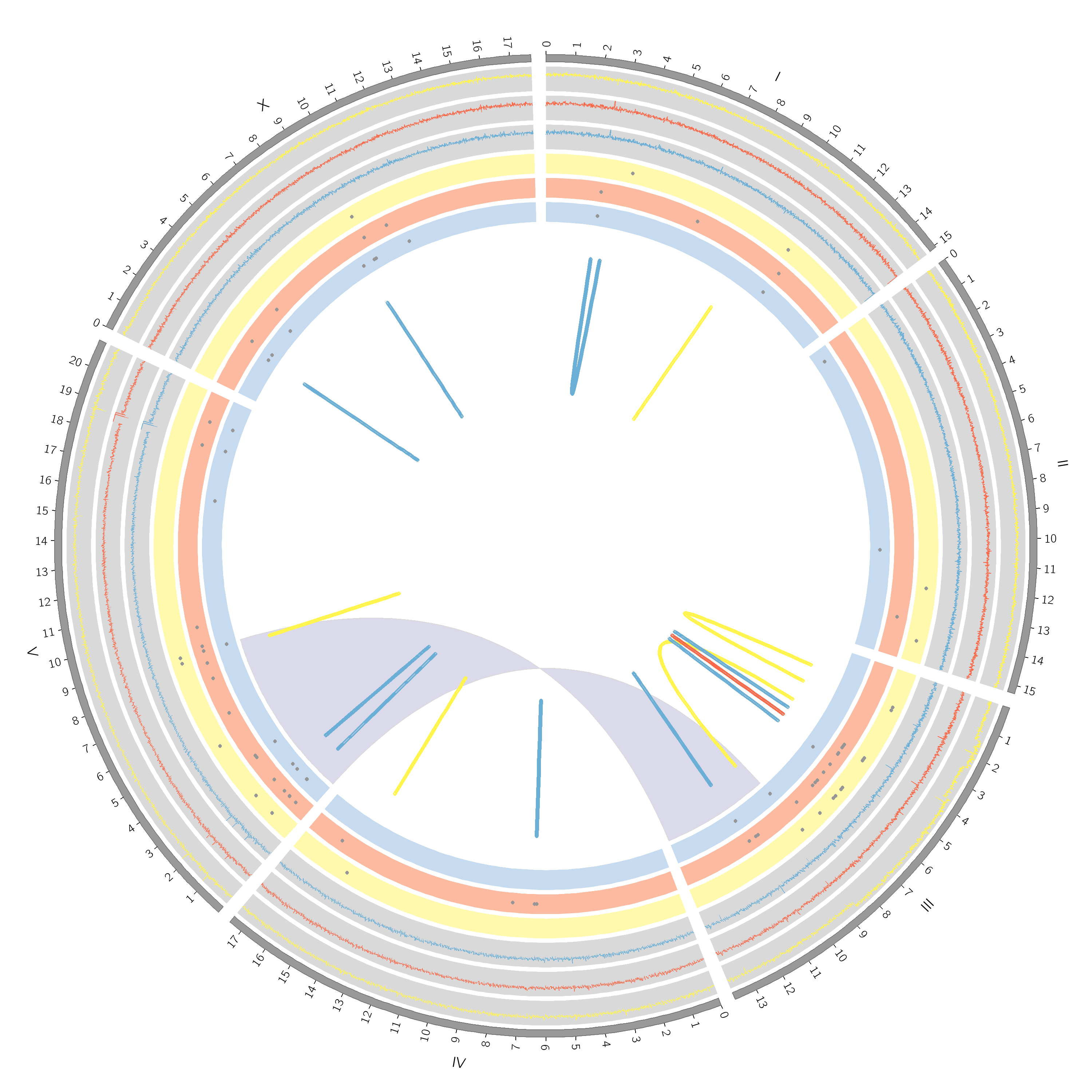

Supplement: Supplementary file 1 — Supplementary Figure 1. [file 41598_2021_97764_MOESM1_ESM.tif]

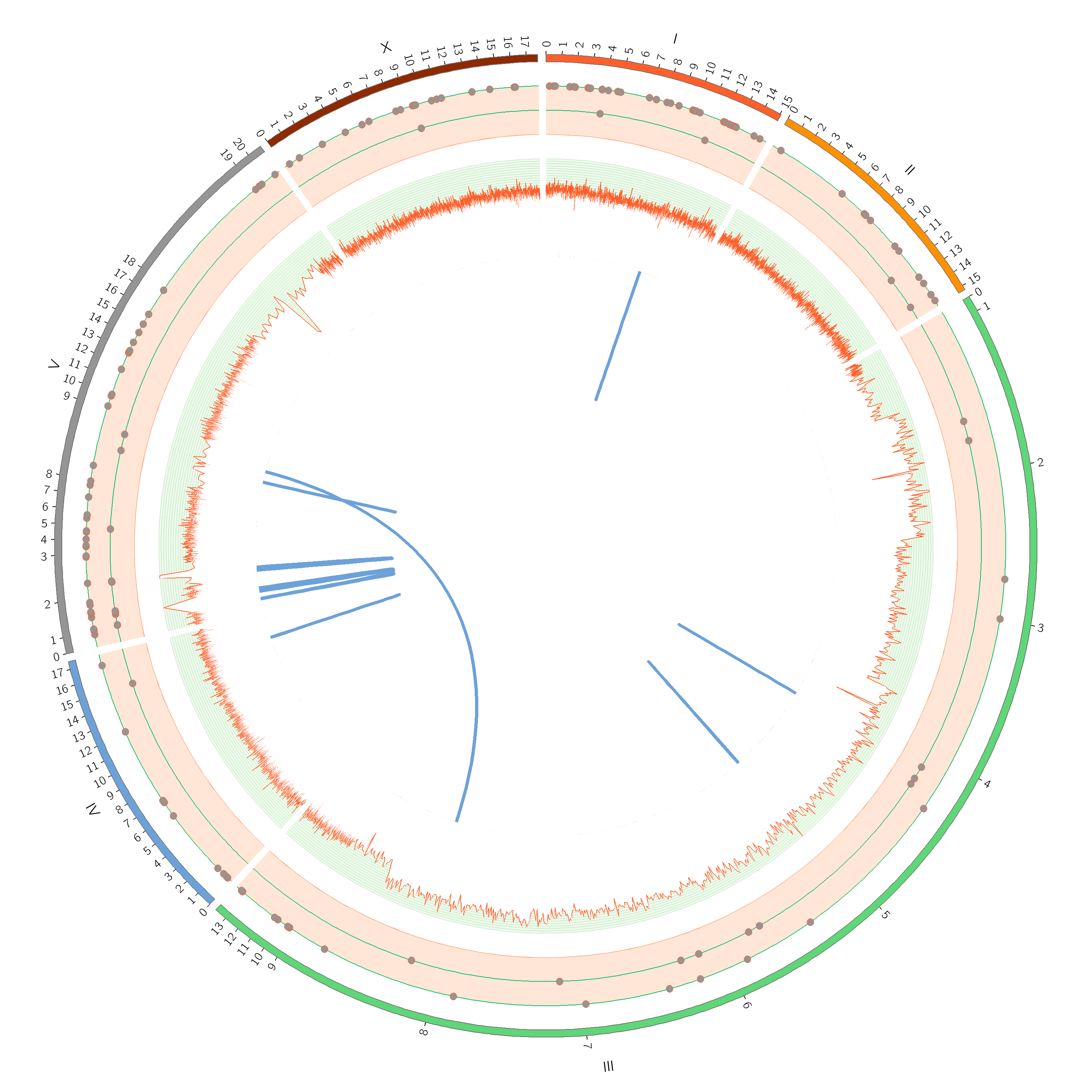

Supplement: Supplementary file 2 — Supplementary Figure 2. [file 41598_2021_97764_MOESM2_ESM.tiff]

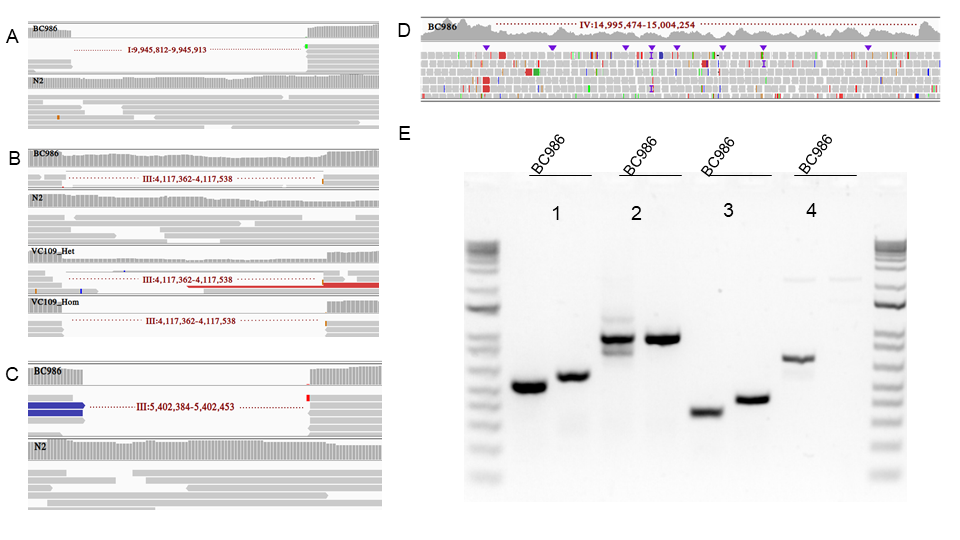

Supplement: Supplementary file 3 — Supplementary Figure 3. [file 41598_2021_97764_MOESM3_ESM.tif]

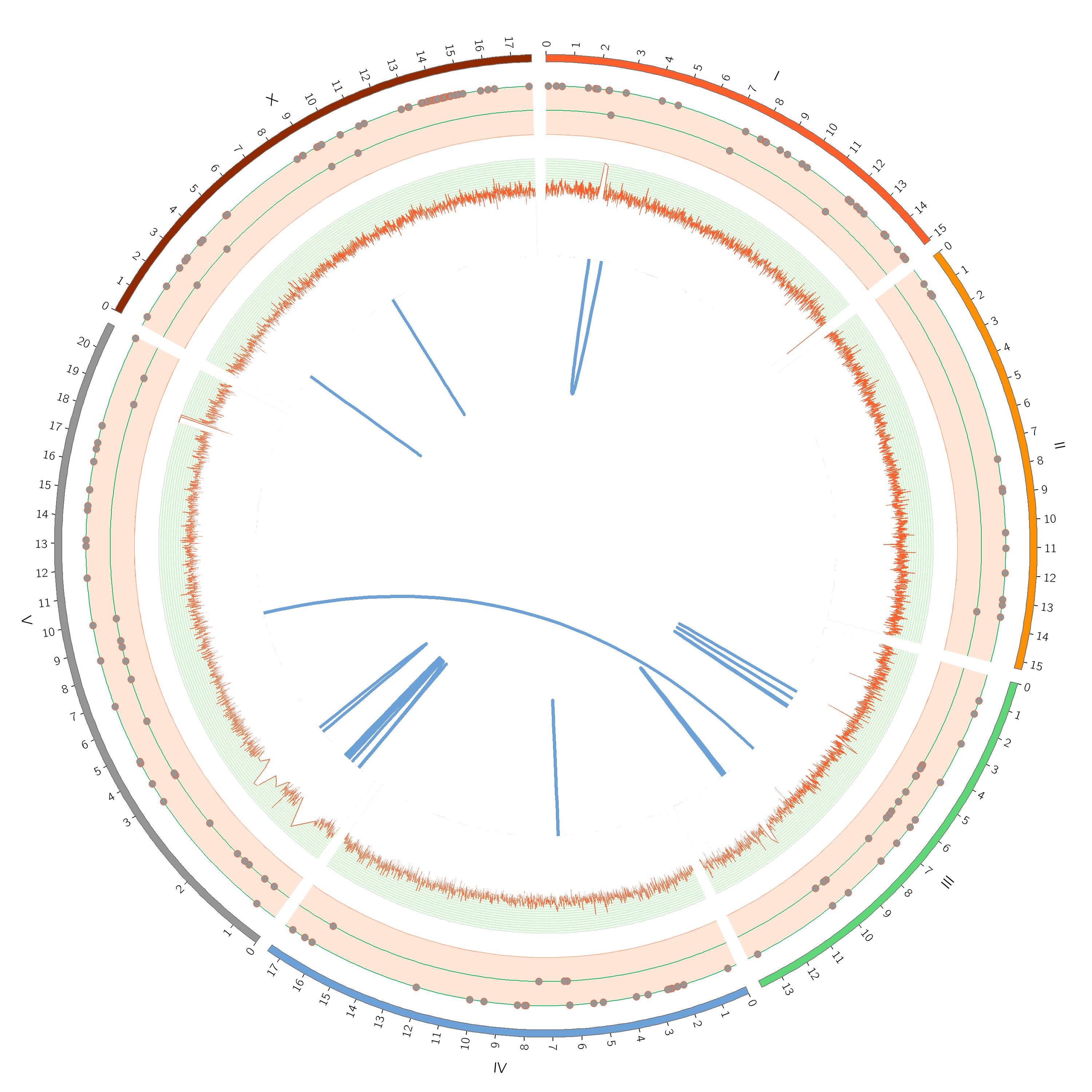

Supplement: Supplementary file 4 — Supplementary Figure 4. [file 41598_2021_97764_MOESM4_ESM.tiff]

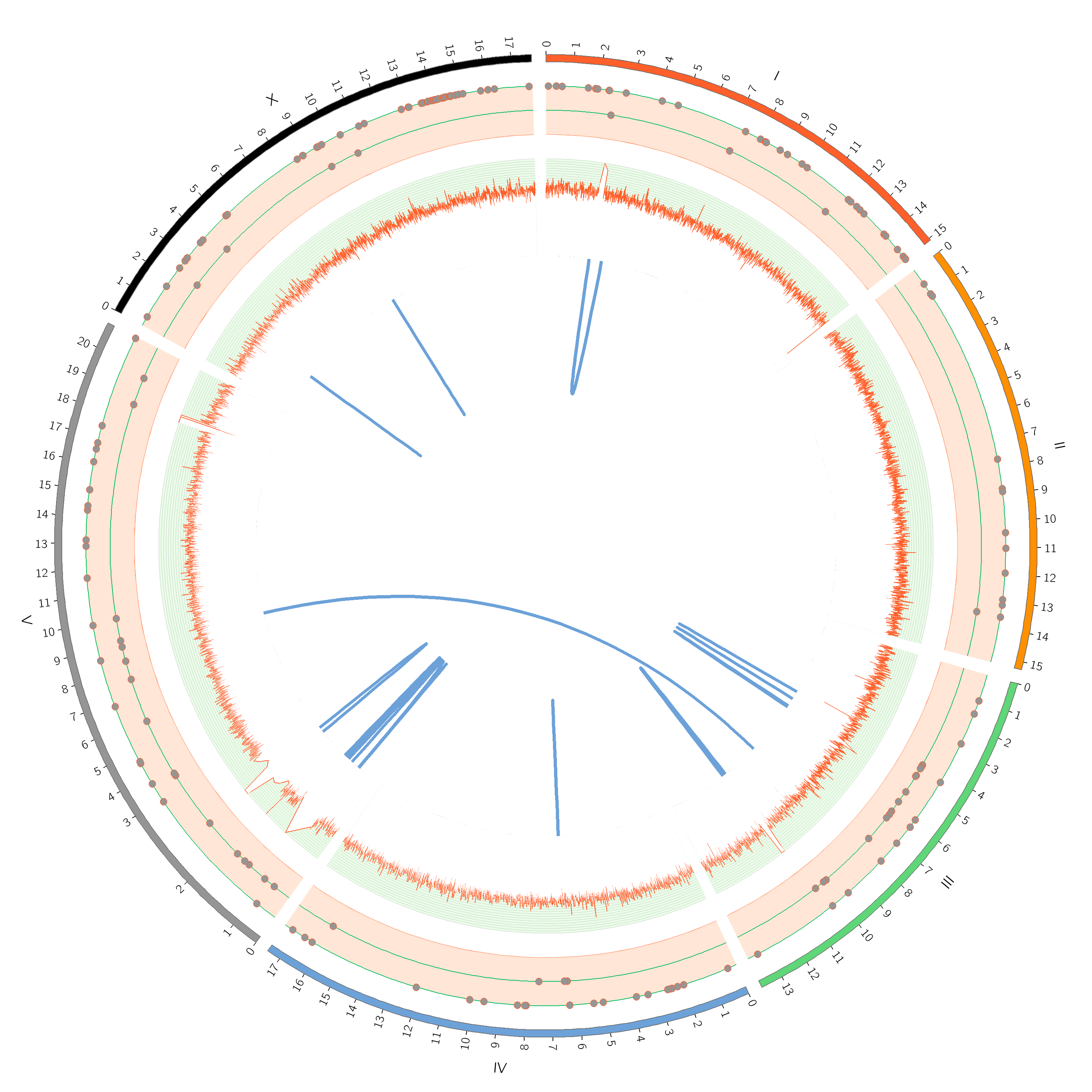

Supplement: Supplementary file 5 — Supplementary Figure 5. [file 41598_2021_97764_MOESM5_ESM.tiff]

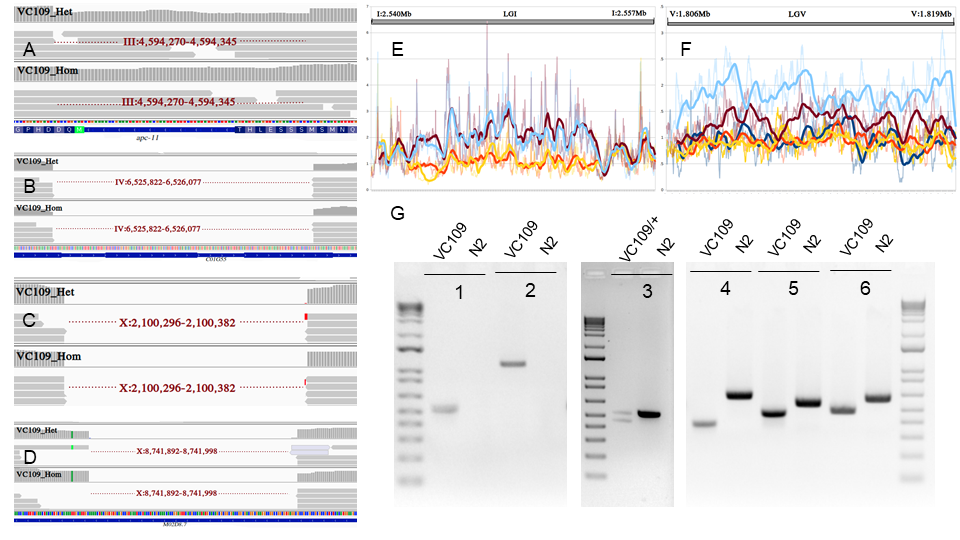

Supplement: Supplementary file 6 — Supplementary Figure 6. [file 41598_2021_97764_MOESM6_ESM.tif]

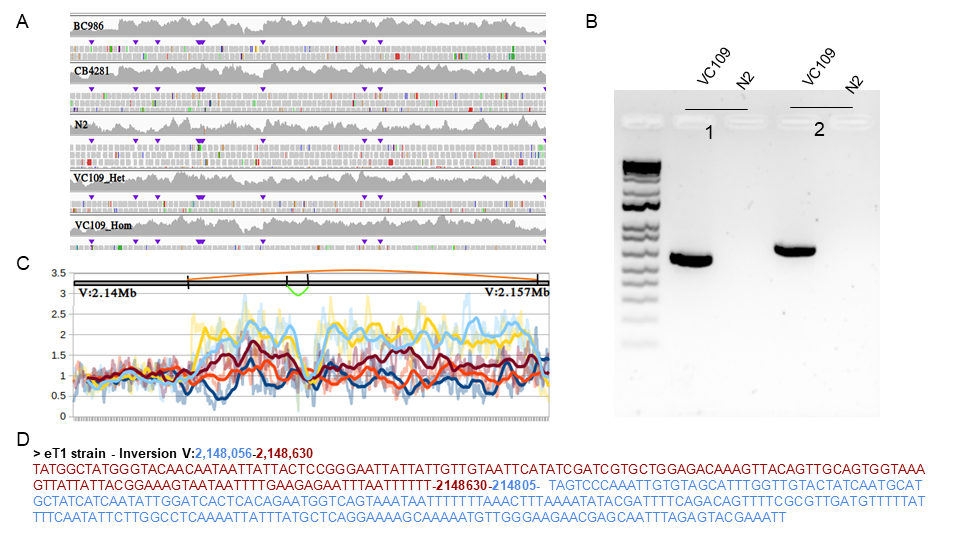

Supplement: Supplementary file 7 — Supplementary Figure 7. [file 41598_2021_97764_MOESM7_ESM.tif]

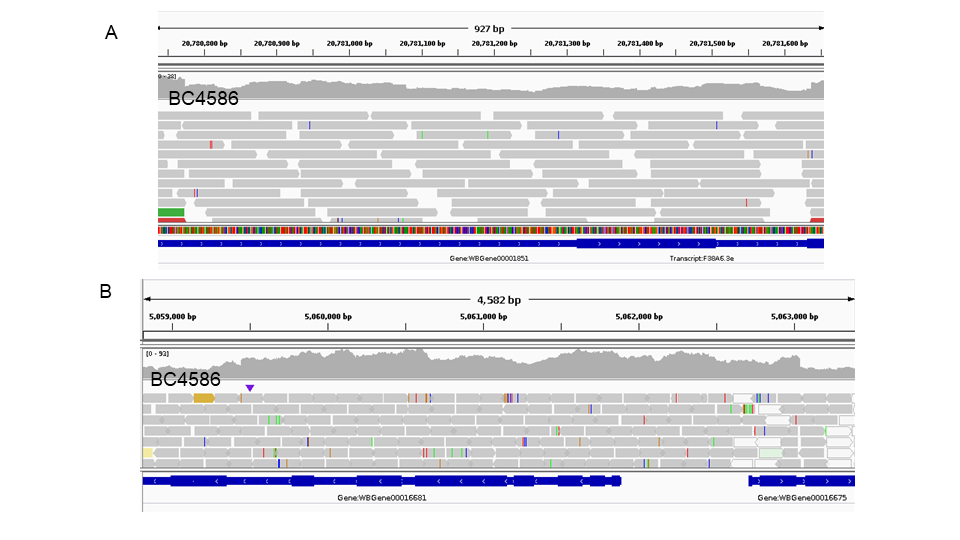

Supplement: Supplementary file 8 — Supplementary Figure 8. [file 41598_2021_97764_MOESM8_ESM.tif]

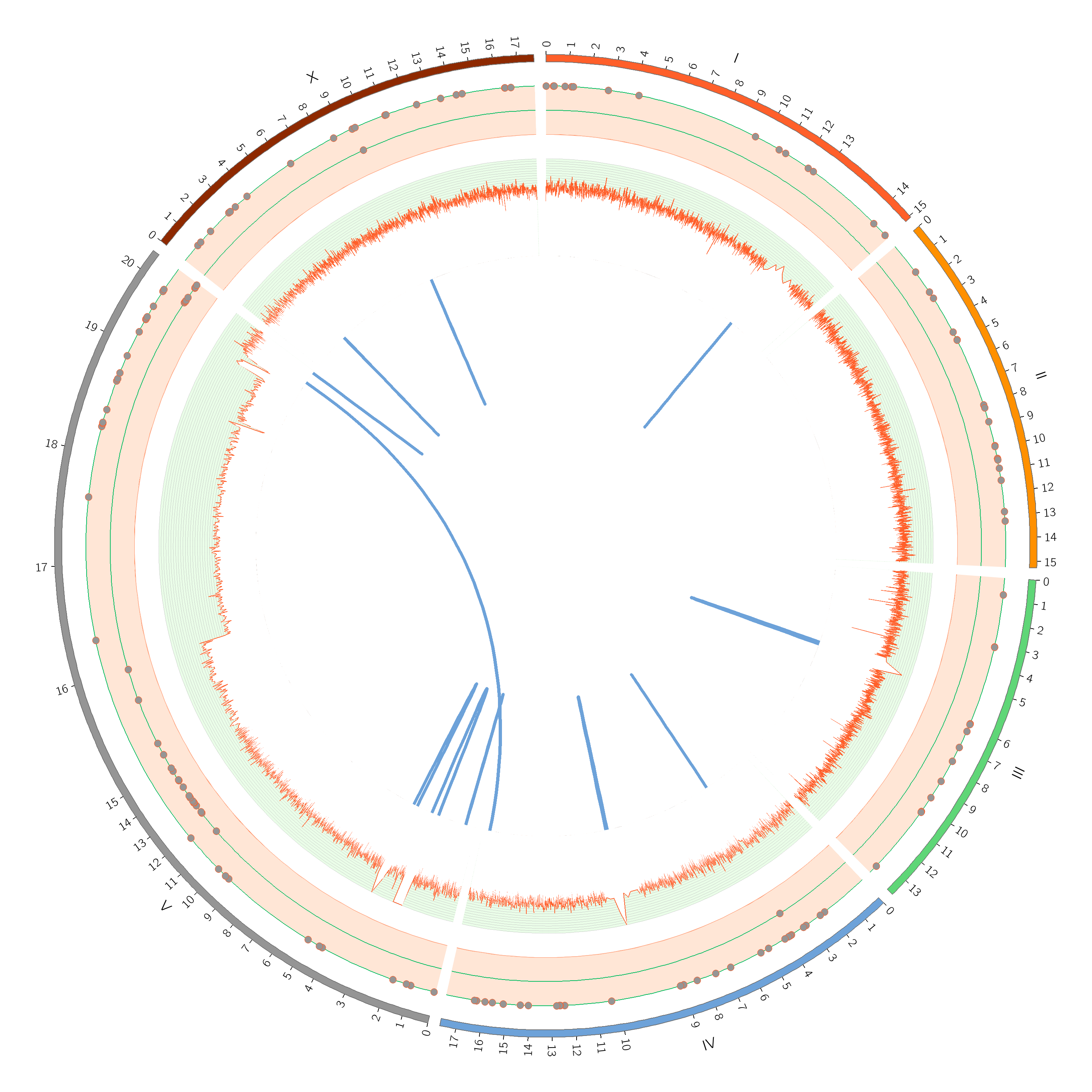

Supplement: Supplementary file 9 — Supplementary Figure 9. [file 41598_2021_97764_MOESM9_ESM.tiff]
