# Supplementary material for: Deciphering complex genome rearrangements in C. elegans using short-read whole genome sequencing
Source: Sci Rep. 2021 Sep 14;11:18258. doi: 10.1038/s41598-021-97764-9 (PMC8440550; doi:10.1038/s41598-021-97764-9)
Supplement: Supplementary file 10 — Supplementary Information. [file 41598_2021_97764_MOESM10_ESM.docx]

# Supplemental information:

**Deciphering complex genome rearrangements in *C. elegans* using short-read whole genome sequencing**

1. **List of Supplementary items:**
2. **Note: Supplemental Tables 1-12 are in a separate file**
3. All Circos plot were produced using Circos (Krzywinski, M. *et al*. 2009)
4. **Supplemental Figure 1.** Circos plots representing *eT1* strains (Figure 1A).

**Supplemental Figure 2.** Circos plots representing SNP genotype, read coverage, and SVs for BC986 strain.

1. **Supplemental Figure 3**. CNVs identified in BC986 genomes.
2. **Supplemental Figure 4.** Circos plots representing SNP genotype, read coverage, and SVs for VC109 strain, wild type looking worms (heterozygous).
3. **Supplemental Figure 5.** Circos plots representing SNP genotype, read coverage, and SVs for VC109 strain, *unc-36* worms (homozygous).
4. **Supplemental Figure 6.** CNVs identified in VC109 genomes.
5. **Supplemental Figure 7.** Inverted duplication part of *eT1* haplotype.
6. **Supplemental Figure 8**. CNVs detected in BC4586.
7. **Supplemental Figure 9.** Circos plots representing SNP genotype, read coverage, and SVs for BC4586 strain.
8. **Supplemental Table 1.** Additional SVs identified in each strain.
9. **Supplemental Table 2.** *eT1* haplotype.
10. **Supplemental Table 3.** Lists of variants called in strain BC986.
11. **Supplemental Table 4**. Sequencing information.
12. **Supplemental Table 5.** Lists of variants called in strain BC4586.
13. **Supplemental Table 6.** Lists of variants called in strain wild-type VC109.
14. **Supplemental Table 7.** Lists of variants called in strain *unc-36* VC109.
15. **Supplemental Table 8.** Primers and Sanger sequencing for *eT1* complex rearrangement on LGV.
16. **Supplemental Table 9.** Primers and Sanger sequencing for VC109 complex rearrangement on LGIII.
17. **Supplemental Table 10.** Primers and Sanger sequencing for BC4586 complex rearrangement on LGIV.
18. **Supplemental Table 11.** Primers and Sanger sequencing for BC986.
19. **Supplemental Table 12.** Raw counts of chromosomes stained with DAPI in N2 and BC4586 oocytes.
20. **Supplemental Figure 1. Circos plot representing *eT1* strains (Figure 1A).** The Circos plot was created with Circos (Krzywinski, M. *et al*. 2009). It displays information regarding BC986 (yellow), wild-type looking VC109 (red; VC109 Het), and *unc-36* VC109 (blue; VC109 Hom) genomes. The outer section is composed of three-line charts (one per strain) representing the ratio of coverage calculated by windows of 1Kb and divided by the strain-specific genome coverage. The middle section is composed of three scatter plots. Each dot represents the genomic position of heterozygous SNPs identified in each strain. The inner section highlights with links and ribbons SVs and complex rearrangements identified in each strain. The *eT1(III;V)* reciprocal translocation is displayed by a blue ribbon.

**Supplemental Figure 2. Circos plot representing SNP genotype, read coverage, and SVs for BC986 strain.** The plot was produced using Circos (Krzywinski, M. *et al*. 2009), The outer section represents the SNPs and indels and their genotype. A dot on the upper line means two alternate alleles (homozygous). A dot on the lower line means one alternate allele (heterozygous). The next section is an orange line chart of the variation of the depth of coverage. The blue links highlight the presence of an SV or complex rearrangement on the genome. Note that the scale is variable. We zoomed in for regions of interest.

**Supplemental Figure 3. CNVs identified in BC986 genomes.** Deletions not described in the main manuscript identified specifically in the strain BC986. A-D) Visualization of the regions surrounding the deletions on Integrative Genome Viewer (IGV) software. A complete absence of reads suggests a homozygous deletion. A) 92 bp homozygous deletions from I:9,945,821 to I:9,945,913. B) 176 bp deletion on LGIII (4,117,362-4,117,538) homozygous in *unc-36* VC109 wormss, heterozygous in wild-type looking VC109 worms, and mosaic in BC986, due to the overlapping with the free duplication *sDp3*. C) 69 bp homozygous deletion on LGIII (5,402,384-5,402,453). D) 8,799 bp heterozygous deletion on LGIV (14,995,474-15,004,254). Average of read depth between the breakpoints = 25.9. Average of read depth over the entire LGIV = 29.7. Average of read depth over BC986 genome = 30.7. 18 supporting reads for the deletion. E) Agarose gel with PCR products for validation of the four deletions detected in BC986 genomes. 1 = homozygous deletion I:9,945,812-9,945,913; 2 = heterozygous deletion III:4,117,362-4,117,538; 3 = homozygous deletion III:5,402,384-5,402,453; 4 = heterozygous deletion IV:14,995,474-15,004,254

**Supplemental Figure 4. Circos plot representing SNP genotype, read coverage, and SVs for VC109 strain, wild-type looking worms (heterozygous).** The plot was created using Circos (Krzywinski, M. *et al*. 2009). The outer section represents the SNPs and indels and their genotype. A dot on the upper line means two alternate alleles (homozygous). A dot on the lower line means one alternate allele (heterozygous). The next section is an orange line chart of the variation of the depth of coverage. The blue links highlight the presence of an SV or complex rearrangement on the genome. Note that the scale is variable. We zoomed in for some regions of interest.

**Supplemental Figure 5. Circos plot representing SNP genotype, read coverage, and SVs for VC109 strain, *unc-36* worms (homozygous).** The plot was created using Circos (Krzywinski, M. *et al*. 2009). The outer section represents the SNPs and indels and their genotype. A dot on the upper line means two alternate alleles (homozygous). A dot on the lower line means one alternate allele (heterozygous). The next section is an orange line chart of the variation of the depth of coverage. The blue links highlight the presence of an SV or complex rearrangement on the genome. Note that the scale is variable. We zoomed in for some regions of interest.

**Supplemental Figure 6. CNVs** **identified in VC109 genomes.**

A-D) Visualization with Integrative Genome Viewer (IGV) software of the regions surrounding deletions detected in VC109. The absence of reads suggests a homozygous deletion. A) Deletion *gk37* (III:4,594,270-4,594,345) previously described for VC109 strain and detected here by short-read WGS. The deletion is heterozygous in wild-type-looking worms (heterozygous for *eT1*). The deletion looks mosaic in *unc-36* worms (homozygous). B) 255 bp homozygous deletion on LGIV (6,525,822-6,526,077). C) 86 bp homozygous deletion on LGX (2,100,296-2,100,382). D) 106 bp homozygous deletion on LGX (8,741,892-8,741,998). E-F) Line charts representing the ratio of the depth of coverage on specific regions of the genome. The ratio of the depth of coverage is calculated by windows of 10bp. For each window we calculated the average of coverage using “samtools depth” and we divided the average by the strain-specific genome coverage. Ratio = 1 means normal coverage (2 copies of the region). Ratio >1 means a copy gain. Ratio <1 means a copy loss. Ratio = 0.5 is a heterozygous deletion (one copy left only). Ratio = 0 is a homozygous deletion (no more copy). Ratio = 1.5 means heterozygous duplication (3 copies). Ratio = 2 means homozygous duplication (4 copies). The faint lines (65% of transparency) represent the raw ratios. The larger line is the trend line representing a moving average of 50 points (500 bp in total). Each color represents a different strain: N2 (dark blue), BC4586 (orange), BC986 (yellow), wild-type VC109 (red) and *unc-36* VC109 (light blue). E) 11,783 bp copy gain on LGI (2,542,068-2,553,851). Homozygous duplication in both VC109 types of worms. F) 12,645 bp copy gain on LGV (1,806,438-1,819,092). Heterozygous direct tandem duplication for wild-type-looking VC109 worms. Homozygous direct tandem duplication for *unc-36* VC109 worms. G) Agarose gel with PCR products for validation of the six VC109 specific CNVs detected. 1 = direct tandem duplication I:2,542,068-2,553,851; 2 = direct tandem duplication I:1,806,438-1,819,092; 3 = deletion III:4,594,270-4,594,345 (*gk37*); 4 = deletion IV:6,525,822-6,526,077; 5 = deletion X:2,100,296-2,100,382; 6 = deletion X:8,741,892-8,741,998

**Supplemental Figure 7. Inverted duplication part of *eT1* haplotype.** A) Visualization with Integrative Genome Viewer (IGV) software of the region surrounding the inverted duplication *eT1* specific on LGV (~2.1 Mb). B) Line charts representing the ratio of the depth of coverage on the region between V:2.14 Mb and V:2.157 Mb for each strain: N2 (dark blue), BC4586 (orange), BC986 (yellow), heterozygous VC109 (red), and homozygous VC109 (light blue). The ratio of the depth of coverage is calculated by windows of 10 bp. For each window we calculated the average of coverage using “samtools depth” and we divided the average by the strain-specific genome coverage. Ratio = 1 means normal coverage (2 copies of the region). Ratio >1 means a copy gain. Ratio <1 means a copy loss. Ratio = 0.5 is a heterozygous deletion (one copy left only). Ratio = 0 is a homozygous deletion (no more copy). Ratio = 1.5 means heterozygous duplication (3 copies). Ratio = 2 means duplication of both copies, so triplication or homozygous duplication (4 copies). The faint lines (65% of transparency) represent the raw ratios. The larger line is the trend line representing a moving average of 50 points (500 bp in total). Copy gain from V:2,144,217 to V:2,156,311. Heterozygous duplication for wild-type-looking VC109 worms. Homozygous duplication for BC986 and *unc-36* VC109 worms. C) Agarose gel with PCR products for validation of copy gain (1) and inversion *eT1* specific (2). D) Sequences of junction regions of the inversion V:2,148,056-2,148,630, causing the drop in the coverage, generated by Sanger sequencing. The sequence was aligned against the *C. elegans* reference genome (WBcel235/ce11) using Blat/UCSC.

**Supplemental Figure 8. CNVs detected in BC4586**. Visualization with Integrative Genome Viewer (IGV) software of the regions surrounding CNVs detected in BC4586. The absence of reads suggests a homozygous deletion. A) 864 bp copy loss (heterozygous deletion) on LGV (20,780,774-20,781,638). B) 3,591 bp copy gain on LGIII. Homozygous direct tandem duplication (ratio of coverage = 2).

1. **Supplemental Figure 9. Circos plot representing SNP genotype, read coverage, and SVs for BC4586 strain**. The plot was produced using Circos (Krzywinski, M. *et al*. 2009), The outer section represents the SNPs and indels and their genotype. A dot on the upper line means two alternate alleles (homozygous). A dot on the lower line means one alternate allele (heterozygous). The next section is an orange line chart of the variation of the depth of coverage. The blue links highlight the presence of an SV or complex rearrangement on the genome. Note that the scale is variable. We zoomed in for regions of interest.
